# The liquid–liquid phase separation signature predicts the prognosis and immunotherapy response in hepatocellular carcinoma

**DOI:** 10.1111/jcmm.18446

**Published:** 2024-07-28

**Authors:** Zhiyong Wang, Guoliang Wang, Peng Zhao, Ping Sun

**Affiliations:** ^1^ Department of Gastrointestinal Surgery, Union Hospital, Tongji Medical College Huazhong University of Science and Technology Wuhan China; ^2^ Department of Hepatobiliary Surgery, Union Hospital, Tongji Medical College Huazhong University of Science and Technology Wuhan China

**Keywords:** hepatocellular carcinoma, immunotherapy efficacy, liquid–liquid phase separation, molecular docking, prognosis

## Abstract

Hepatocellular carcinoma (HCC) is a common and fatal malignancy characterized by poor patient prognosis and treatment outcome. The process of liquid–liquid phase separation in tumour cells alters the dysfunction of biomolecular condensation in tumour cells, which affects tumour progression and treatment. We downloaded the data of HCC samples from TCGA database and GEO database, and used a machine learning method to build a new liquid–liquid phase separation index (LLPSI) by liquid–liquid phase separation related genes. The LLPSI‐related column line Figure was constructed to provide a quantitative tool for clinical practice. HCC patients were divided into high and low LLPSI groups based on LLPSI, and clinical features, tumour immune microenvironment, chemotherapeutic response, and immunotherapeutic response were systematically analysed. LLPSI, which consists of five liquid–liquid phase separation‐associated genes (MAPT, WDR62, PLK1, CDCA8 and TOP2A), is a reliable predictor of survival in patients with HCC and has been validated in multiple external datasets. We found that the high LLPSI group showed higher levels of immune cell infiltration and better response to immunotherapy compared to the low LLPSI group, and LLPSI can also be used for prognostic prediction in various cancers other than HCC. In vitro experiments verified that knockdown of MAPT could inhibit the proliferation and migration of HCC. The LLPSI identified in this study can accurately assess the prognosis of patients with HCC and identify patient populations that will benefit from immunotherapy, providing valuable insights into the clinical management of HCC.

## 1 BACKGROUND

Hepatocellular carcinoma (HCC) is one of the most common types of cancer and is also the third leading cause of cancer‐related deaths.^1^ HCC is the predominant liver cancer subtype and accounts for 80% of the total liver cancer burden worldwide.^2^ Currently, several therapeutic strategies, such as surgical resection, liver transplantation, localized therapy, and systemic therapy, have been shown to be effective in controlling HCC, but these treatment modalities fail to provide satisfactory results due to factors such as postoperative recurrence, treatment resistance, and difficulty in early diagnosis.^3^ The mechanisms of HCC progression have been extensively studied but are not yet fully understood. In this context, further exploration of novel biomarkers and therapeutic indicators that can reliably predict the prognosis of HCC is of great significance for the prevention and treatment of HCC.

An increasing number of studies have shown that liquid–liquid phase separation (LLPS), which can form various membrane‐free compartments, is an important anti‐cancer defence mechanism and therapeutic target.^4^ However, few studies have considered liquid–liquid phase separation from the perspective of tumour immunotherapy. Liu et al. found that circASH2‐enhanced liquid–liquid phase separation‐mediated cytoskeletal remodelling of nuclear Y box‐binding protein 1 (YBX1) inhibited HCC metastasis.^5^ The above findings emphasize the need to investigate potential clinical targets in HCC from a liquid–liquid phase separation perspective.

In this study, we established a new index, the liquid–liquid phase separation index (LLPSI), by collecting genes during the liquid–liquid phase separation process and thus by a machine learning approach to predict the prognosis of HCC patients and the effectiveness of therapeutic interventions. We performed in vitro experimental assays to evaluate the role of MAPT in HCC progression.

## 2 MATERIALS AND METHODS

### 2.1 Data sources and preprocessing

We downloaded gene expression, immune infiltration and corresponding clinical information of 374 HCC patients and 50 normal liver tissue cases from the TCGA database (https://portal.gdc.cancer.gov/).^6^ Key genes expressing liquid–liquid phase separation proteins were collected as LLPS‐related genes from the DrLLPS database (http://llps.biocuckoo.cn/)^7^ and review articles. In addition, two HCC‐related datasets, GSE45267 and GSE121248, were obtained from the GEO database (https://www.ncbi.nlm.nih.gov/geo/) as validation cohorts. Expression analysis and visualization were performed by the ‘ggplot2’ package in R software. The Human Protein Atlas (HPA) (https://www.proteinatlas.org/) is a comprehensive public database of human protein information, mainly used to analyse the expression of specific proteins.

### 2.2 Construction and validation of LLPSI

The training dataset (TCGA samples) was used to construct LLPSI‐related features to predict the prognosis of HCC patients. We obtained five LLPS‐related genes with the best prognostic values and constructed LLPSIs by analysis of differences between normal liver tissue and HCC tissue (LogFc >2, fdr <0.05), by prognostic analysis of HCC (*p* < 0.01), and by univariate cox regression analysis and multivariate Cox analysis. The risk score formula for each sample was as follows: LLPSI = Coef (Gene 1) × Expr (Gene 1) + Coef (Gene 2) × Expr (Gene 2) +…+ Coef (Gene n) × Expr (Gene n) where, Coef (Gene) represents the risk regression coefficient of Gene and Expr(Gene) represents the expression of Gene. Based on the median value of LLPSI, we categorized HCC patients into high LLPSI and low LLPSI groups.

### 2.3 Analysis of the immune microenvironment

The CIBERSORT method was used to quantify the relative proportions of infiltrating immune cells (https://cibersort.stanford.edu/).^8^ The proportions of 22 immune cells were calculated by the CIBERSORT method (B naïve cells, B cell memory, plasma cells, T cell CD8, T cell CD4 naïve, T cell follicular helper cells, T cell CD4 memory resting, T cell CD4 memory activated, regulatory T cells (Tregs), γ δ cells, monocytes, activated NK cells, resting NK cells, Macrophage M0, Macrophage M1, Macrophage M2, Resting Dendritic Cells, Activated Dendritic Cells, Resting Mast Cells, Activated Mast Cells, Eosinophils, and Neutrophils). *p* < 0.05 samples indicate that the proportions of immune cells calculated by CIBERSORT are correct. Tumour purity, stromal score, immune score and ESTIMATE score were calculated for each tumour sample by the R package ‘ESTIMATE’.^9^ The Single Sample Gene Set Enrichment Analysis (ssGSEA) algorithm was used to assess the immune infiltration between the two groups based on 28 immune cell types. The relative proportion of immune cell infiltration was also quantified by XCELL, QUANTISEQ, MCPCOUNTER, EPIC and CIBERSORT‐ABS software.

### 2.4 Drug therapy response and immunotherapy response

The GDSC database (Genomics of Drug Sensitivity in Cancer) (https://www.cancerrxgene.org/)^10^ was utilized to predict chemotherapy response for each sample. The Tumour Immune Dysfunction and Exclusion (TIDE) algorithm can be used to infer patient response to immunotherapy.^11^

### 2.5 Molecular docking simulation

We used Schrödinger software to screen small‐molecule drugs that bind to target proteins and perform molecular docking simulations. We downloaded the protein structures of the target targets (PLK1‐5TA8 and TOP2A‐4R1F) from the PDB database and the structures of the natural small molecule drugs from the PubChem database (https://pubchem.ncbi.nlm.nih.gov/). The binding poses of PLK1 and TOP2A with small molecule drugs were simulated by the molecular docking module in Schrödinger software.

### 2.6 Bioinformatics analysis

Survival curves were plotted using the ‘survival’ package. The ‘pROC’, ‘timeROC’ and ‘ggrisk’ packages were used to perform the ROC, time‐dependent ROC and risk factor analyses, respectively. Risk factor analyses were performed and the results were visualized using the ‘ggplot2’ software package.

### 2.7 Small interfering RNA (siRNA) transfection

HepG2 and Huh7 cells were inoculated in cell culture plates and MAPT was knocked down using siRNA according to the kit instructions.

### 2.8 EdU cell proliferation assay

The si‐MAPT‐transfected HepG2 and Huh7 cells were inoculated in 24‐well plates and each well was incubated with EdU medium for 2 h. The cells were washed twice with PBS. Cell fixation solution (PBS containing 4% paraformaldehyde) was added to each well and fixed at room temperature for 30 min, after which the glycine decolorization shaker was added and incubated for 5 min before discarding the glycine solution and adding 100 μL of permeant decolorization shaker with slow shaking permeabilization. After that, staining reaction solution was added to each well under light‐proof treatment.

### 2.9 Transwell experiments

Trypsin digestion of cells and preparation of cell suspension. The cells were resuspended with serum‐free medium, counted on cell counting plates, and the complete medium was pre‐filled in 24‐well plates and put into transwell chambers. 1 h later, each group of cell suspensions was inserted into the upper chamber of the transwell, and the cells were incubated in a 5% CO2 incubator at 37°C for 24 h; the cells were stained with 0.5% crystal violet staining solution and the staining solution was left at room temperature for 20 min, and then observed and photographed under a microscope.

### 2.10 Wound‐healing experiments

Trypsin digested the cells and prepared cell suspension. Cells were counted and spread on the plate, and a 10 uL gun was used to make cell scratches, the medium was aspirated and the cells were washed gently with PBS for three times to fully remove the floating cells, and the cells were taken out and photographed under the microscope after 48 h.

### 2.11 Statistical analysis

Survival curves were plotted using the Kaplan–Meier method to compare the survival differences between the two groups. Receiver Operation Characteristic (ROC) curves, one‐way and multifactorial Cox analyses were used to assess the prognostic value of the characteristics. Spearman correlation analysis was used to assess correlation. *p*≤0.05 was considered statistically significant. All statistical analyses were performed by R.

## 3 RESULTS

### 3.1 Unsupervised clustering of liquid–liquid phase separation‐related genes in HCC

**FIGURE 1:**
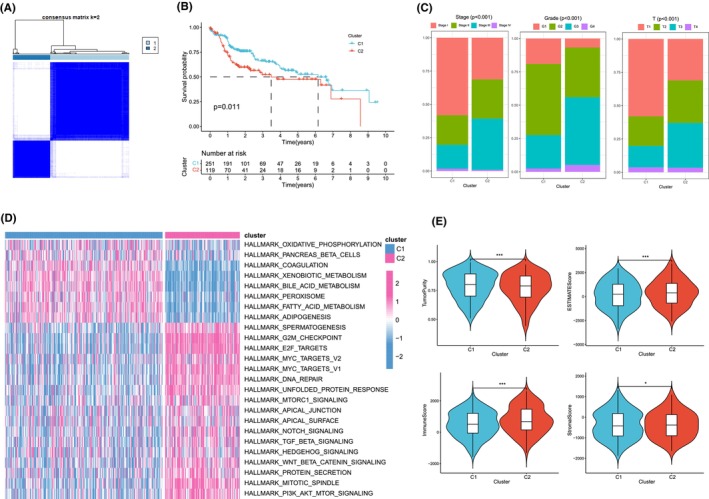
Unsupervised cluster analysis of programmed cell death genes. (A) When *k* = 2, hepatocellular carcinoma patients were divided into two clusters based on programmed cell death‐related genes. (B) Kaplan–Meier curves showing the prognosis of hepatocellular carcinoma patients in both clusters. (C) The ratio of clinicopathologic features between the two clusters. (D) Heatmap of enrichment in the HALLMARK pathway between the two clusters found by enrichment analysis. (E) Differences in tumour purity, ESTIMATEScore, immune score and stroma score between the two clusters. Note *p < 0.05, **p < 0.01, ***p < 0.001.

We performed cluster analysis to explore HCC subtypes by collecting LLPS‐related genes. We found that differences between subgroups were most significant when *k* = 2, indicating that HCC patients could be well categorized into two clusters (Figure 1A). We found a significant difference in patient overall survival (OS) between the two clusters (*p* < 0.05), with C1 associated with patients with a good prognosis and C2 associated with patients with a poor prognosis (Figure 1B). By comparing the differences in clinicopathologic features between the two clusters, we found that the percentage of clinical stage, grading and T stage were significantly higher in C2 than in C1, suggesting that the malignant phenotype was higher in C2 patients (Figure 1C). Subsequently, we investigated the enriched pathways between the two clusters by gene set enrichment analysis, which showed that Cluster1 was enriched in the pathways fatty acid metabolism, coagulation, and bile acid metabolism, and Cluster2 was enriched in G2M CHECKPOINT, MTORC1 signalling, notch signalling and PI3K AKT MTOR SIGNALLING pathways (Figure 1D). We then analysed the immune profile between the two clusters. We found that the tumour purity of C2 was significantly lower than that of C1, while the ESTIMATEScore, immunity score and stroma score of C2 were significantly higher than that of C1, suggesting a high level of immune infiltration in the C2 type compared to C1 (Figure 1E).

### 3.2 Construction of liquid–LLPSI in HCC patients

We identified five LLPS‐related genes by one‐way Cox regression and multifactorial Cox regression analyses and then constructed the LLPSI derived from liquid–liquid phase separation.Our model derived the LLPSI for each patient by the following equation: LLPSI = (0.1932 × CDCA8 exp.) + (0.2802 × PLK1 exp.) + (−0.0797 × TOP2A exp.) + (−0.3575 × WDR62 exp.) + (0.2655 × MAPT exp.).

### 3.3 External dataset validation and clinical relevance of LLPSI

**FIGURE 2:**
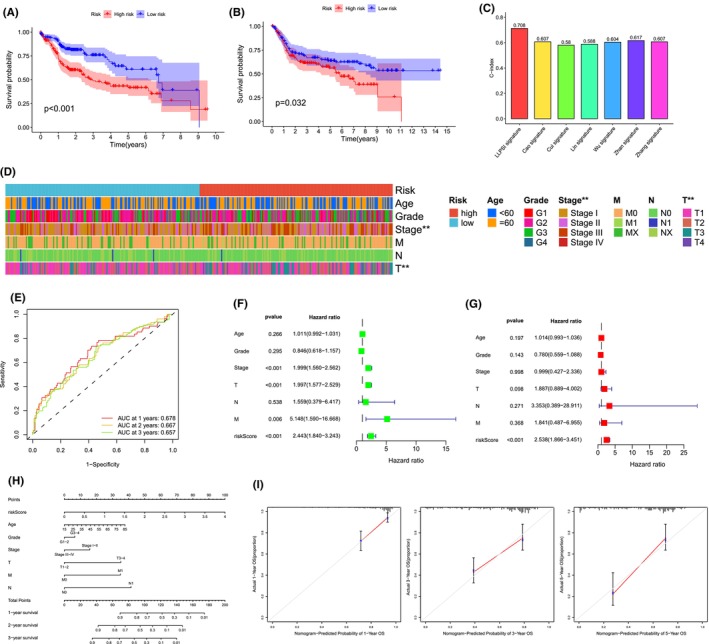
External dataset validation and clinical relevance of LLPSI. kaplan–Meier survival curves comparing the TCGA dataset (A) and the GEO external dataset (B) for high/low LLPSI. (C) Comparison of LLPSI index with other prognostic models for hepatocellular carcinoma. (D) Heatmap of correlation between common clinical features and LLPSI. (E) ROC curves for LLPSI at 1, 3 and 5 years. Univariate (F) and multivariate (G) Cox regression analysis of LLPSI and other clinical traits. (H) Column line graph predicting prognosis in patients with hepatocellular carcinoma. (I) Calibration curves for the probability of 1‐, 3‐, and 5‐year overall survival for column‐line plots in the TCGA cohort.

We found that patients in the high LLPSI group had a worse prognosis and those in the low LLPSI group had a better prognosis by using the Kaplan–Meier survival curves, suggesting that patients in the high LLPSI group had a higher mortality rate (Figure 2A). Subsequently, we used the GSE45267 and GSE121248 integration datasets as the validation cohort. As with the results of the training cohort, patients with HCC in the high LLPSI group had poorer OS (Figure 2B). We collected published features of prognostic models for HCC and compared the LLPSI features of this study with their prognostic prediction accuracy, and showed that LLPSI values outperformed other models in terms of accuracy in prognostic prediction for patients with HCC. (Figure 2C).^12^, ^13^, ^14^, ^15^, ^16^, ^17^

To further validate the clinical significance of LLPSI, we analysed the association between LLPSI and different clinical traits and found that LLPSI was significantly associated with patients' Stage and T‐stage (Figure 2D). We evaluated the area under the curve (AUC) values and showed that LLPSI had high accuracy in predicting 1‐, 3‐ and 5‐year survival in patients with HCC (Figure 2E). The results of univariate and multivariate Cox regression analyses showed that LLPSI could be used as an independent prognostic factor to predict the prognosis of HCC patients (Figure 2F,G). After that, we established a column‐line diagram model for HCC patients to assess the patients' prognosis, and age, grade, clinical stage, T, N, and M were included in the column‐line diagram model (Figure 2H). The calibration curves of the column‐line plots showed that the predicted 1‐, 3‐ and 5‐year survival rates of HCC patients were more consistent with the actual survival rates of the reference line, indicating that the constructed column‐line plots could predict the prognosis of HCC patients well (Figure 2I).

### 3.4 LLPSI predicts pan‐cancer prognosis

**FIGURE 3:**
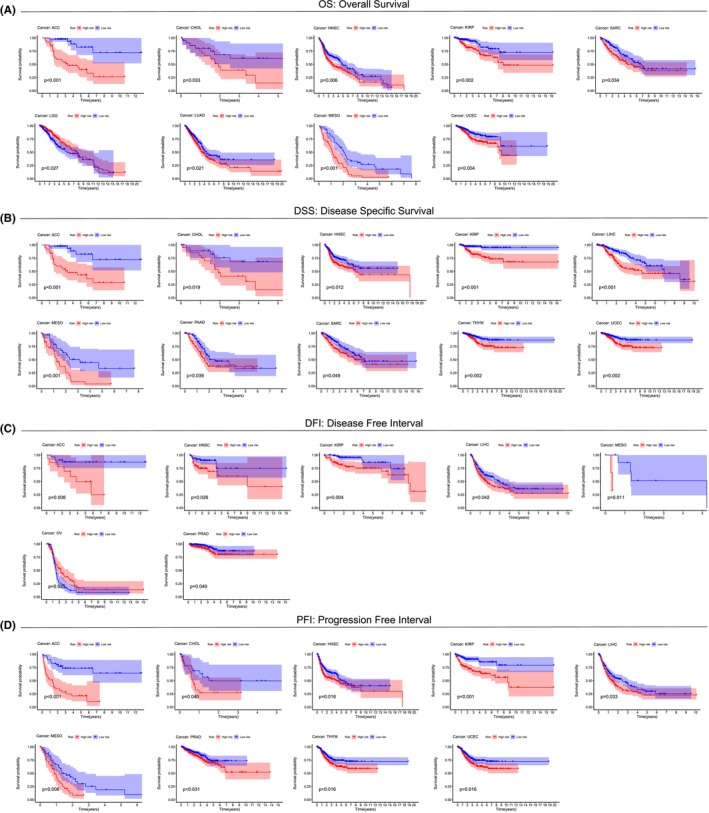
Predictive value of LLPSI in other cancers. (A) OS survival curves of patients in the high/low LLPSI group in ACC, CHOL, HNSC, KIRP, SARC, LGG, LUAD, MESO and UCEC. (B) DSS survival curves of patients in the high/low LLPSI group in ACC, CHOL, HNSC, KIRP, LIHC, MESO, PAAD, SARC, THYM and UCEC. (C) DFI survival curves of patients in the high/low LLPSI group in ACC, HNSC, KIRP, LIHC, MESO, OV and PRAD. (D) PFI survival curves of patients in the high/low LLPSI group in ACC, CHOL, HNSC, KIRP, LIHC, MESO, PRAD, THYM and UCEC.

To further investigate the value of LLPSI in prognostic prediction of other cancer patients, we used the above modelling formula for LLPSI to calculate LLPSI for other cancer patients and plot survival curves for the high LLPSI and low LLPSI groups. Firstly, we found that LLPSI not only predicted OS, Disease Specific Survival (DSS), Disease Free Interval (DFI) and Progression Free Interval (PFI), and also predicted OS, DSS, DFI, and PFI in patients with ACC, HNSC, KIRP and MESO.In addition, for OS, patients in the high LLPSI group had a worse prognosis in CHOL, SARC, LUAD and UCEC, whereas patients in the low LLPSI group had a worse prognosis in LGG (Figure 3A). For DSS, patients in the high LLPSI group had a worse prognosis in CHOL, PAAD, SARC, THYM and UCEC compared with patients in the low LLPSI group (Figure 3B). For DFI, STAD and KIRP, patients in the high LLPSI group had a poor prognosis compared to patients in the low LLPSI group. While in CHOL, PRAD and SARC, patients in the high LLPSI group had a better prognosis (Figure 3C). For PFI, in CHOL, PRAD, THYM and UCEC, patients in the high LLPSI group had a poor prognosis compared with patients in the low LLPSI group (Figure 3D).

### 3.5 LLPSI‐based tumour microenvironment dissection in HCC

**FIGURE 4:**
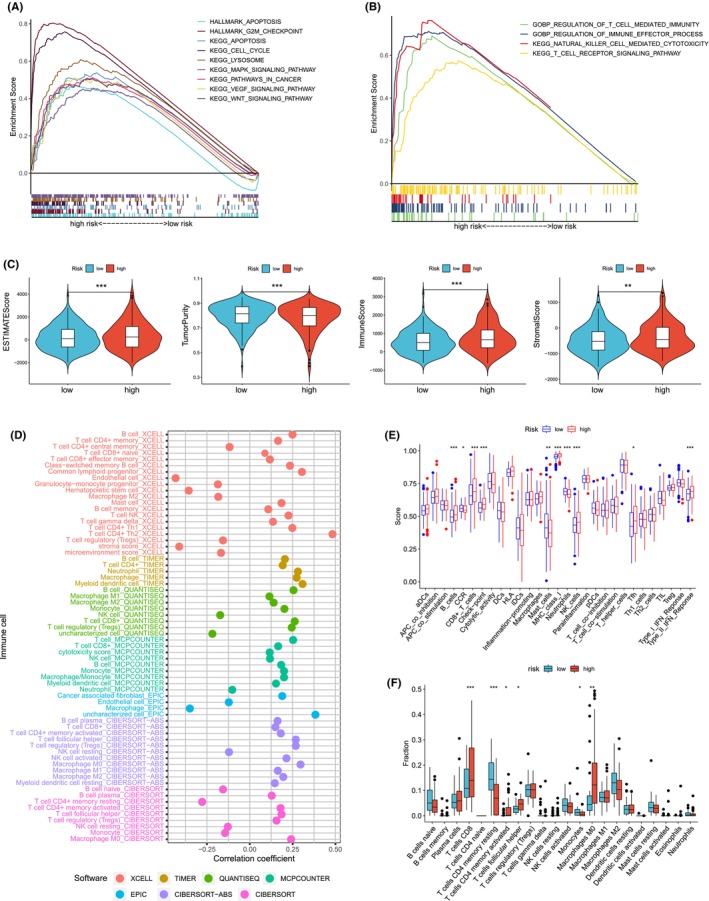
Assessment of tumour microenvironment based on liquid–liquid phase separation index. (A, B) GSEA analysis of patients in the high LLPSI group. (C) Differences in tumour purity, ESTIMATEScore, immunoscore and stromal score between high/low LLPSI groups. (D) XCELL and CIBERSORT software to analyse the correlation between LLPSI and immune cell infiltration. (E) The ssGSEA algorithm assessed the differences in immune cells and immune function between patients in the high/low LLPSI group. (F) CIBERSORT algorithm to assess immune cell differences between patients in the high/low LLPSI group. Note *p < 0.05, **p < 0.01, ***p < 0.001.

In order to investigate the regulatory pathways of tumorigenesis in the high LLPSI group, we performed GSEA analysis on the data of HCC samples, and the results showed that the high LLPSI group was significantly enriched in APOPTOSIS, G2M checkpoint, cell cycle, lysosome, mapk signalling pathway, pathways in cancer, VEGF signalling pathway and wnt signalling pathway signalling pathways were significantly enriched (Figure 4A). In addition, tumours in the high LLPSI group were significantly enriched in regulation of *t* cell mediated immunity, regulation of immune effector process, natural killer cell mediated immunity and *t* cell receptor signalling pathway were all significantly enriched in immunofunctional pathways, suggesting that our high LLPSI group was closely associated with the immune microenvironment (Figure 4B).

The ESTIMATE algorithm revealed that the high LLPSI group had lower tumour purity but high ESTIMATE score, immune score, and stroma score compared with the low LLPSI group (Figure 4C). We found a correlation between LLPSI and different immune cell infiltration, such as CD8 T cells and CD4 T cells, by XCELL, TIMER, QUANTISEQ, MCPCOUNTER, EPIC, CIBERSORT‐ABS, and CIBERSORT software (Figure 4D). ssGSEA algorithm results showed that compared to the low LLPSI group, patients in the high LLPSI group had better immune cell infiltration and immune‐related functions, for example, the high LLPSI group had high levels of immune checkpoint levels, B cells and CD8 T cell infiltration (Figure 4E).The CIBERSORT algorithm showed that the level of immune‐stimulated CD8 T cells in the high PCDI group was significantly higher than that of the low PCDI group, whereas the monocyte levels were significantly lower (Figure 4F).

### 3.6 Efficacy of LLPSI in predicting immunotherapy outcome

**FIGURE 5:**
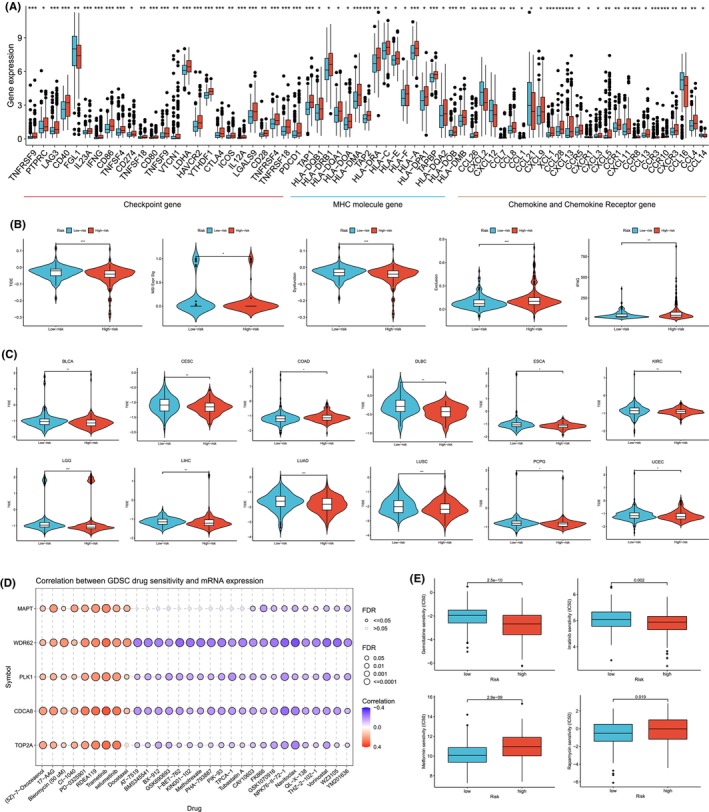
Efficacy of LLPSI in predicting immunotherapy efficacy and drug sensitivity. (A) Differences in the expression levels of common immune checkpoints, MHC molecules and cytokines and their receptors between the high and low LLPSI groups. (B) Differences between TIDE scores, MSI, Dysfunction, Exclusion and IFNG between the high and low LLPSI groups. (C) LLPS score is used for immunotherapy efficacy in pan‐cancer. (D) The GDSC database analysed the relationship between drug sensitivity and mRNA expression of five genes that construct LLPSI. (E) Differences in response to common chemotherapeutic agents between high and low LLPSI groups. Note *p < 0.05, **p < 0.01, ***p < 0.001.

To further explore the relationship between LLPSI and the immune microenvironment, we compared the differences in the expression levels of common immune checkpoints, MHC molecules, and cytokines and their receptors between the high/low LLPSI groups, and the results showed that most of the immune checkpoints, MHC molecules and cytokines and their receptors in patients in the high‐LLPSI group were significantly higher than those in the low‐LLPSI group (Figure 5A). By the results of immune microenvironment analysis, we learned that the high LLPSI group had high immune infiltration characteristics. Combined with the results of immune checkpoints, the tumours in the high LLPSI group showed the characteristics of ‘hot’ tumours, so it can be inferred that the patients in the high LLPSI group may be more effective for immunotherapy than those in the low LLPSI group.

Patients with lower TIDE scores were more likely to benefit from immunotherapy.^11^ Compared with patients in the low LLPSI group, patients in the high LLPSI group had lower TIDE scores and Dysfunction and higher MSI, Exclusion, and IFNG, suggesting that patients in the high LLPSI group had better efficacy for immunotherapy (Figure 5B). We also applied the LLPSI score in pan‐cancer to evaluate its value in predicting immunotherapy outcomes in other cancers, and we found that LLPSI not only predicted HCC immunotherapy efficacy well, but also predicted immunotherapy outcomes in a variety of cancers, such as in BLCA, CESC, COAD, DLBC, ESCA, KIRC, LGG, LIHC, LUAD, LUSC, PCPG and UCEC, patients in the high LLPSI group may have higher immunotherapy efficacy (Figure 5C). All these results indicated that LLPSI could better predict the immunotherapy effect in HCC, and the high LLPSI group had better effect on immunotherapy.

### 3.7 Efficacy of LLPSI in predicting drug sensitivity

To explore the association between LLPSI and drug sensitivity, we analysed the relationship between drug sensitivity and mRNA expression of five genes (MAPT, WDR62, PLK1, CDCA8 and TOP2A) constructing LLPSI using the GDSC database, and a positive correlation indicated that the gene expression was correlated with drug resistance, whereas a negative correlation indicated that the gene expression was correlated with drug sensitivity. The results indicated that MAPT, WDR62, PLK1, CDCA8 and TOP2A gene expression was associated with most drug sensitivities (Figure 5D). We calculated half maximal inhibitory concentration (IC50) values for common drugs in HCC samples and compared them with those between LLPSI subgroups. We found that many of the common chemotherapeutic drugs for HCC were significantly different between the high/low LLPSI groups (*p* < 0.05), and the IC50 values of Gemcitabine and Imatinib were significantly lower in the high LLPSI group than in the low LLPSI group, suggesting that patients in the high LLPSI group may have a better response. The IC50 values for Metformin and Rapamycin were lower in the low LLPSI group compared to the high LLPSI group, suggesting that patients in the low LLPSII group had a better response to Metformin and Rapamycin‐based chemotherapy (Figure 5E).

### 3.8 Molecular docking

**FIGURE 6:**
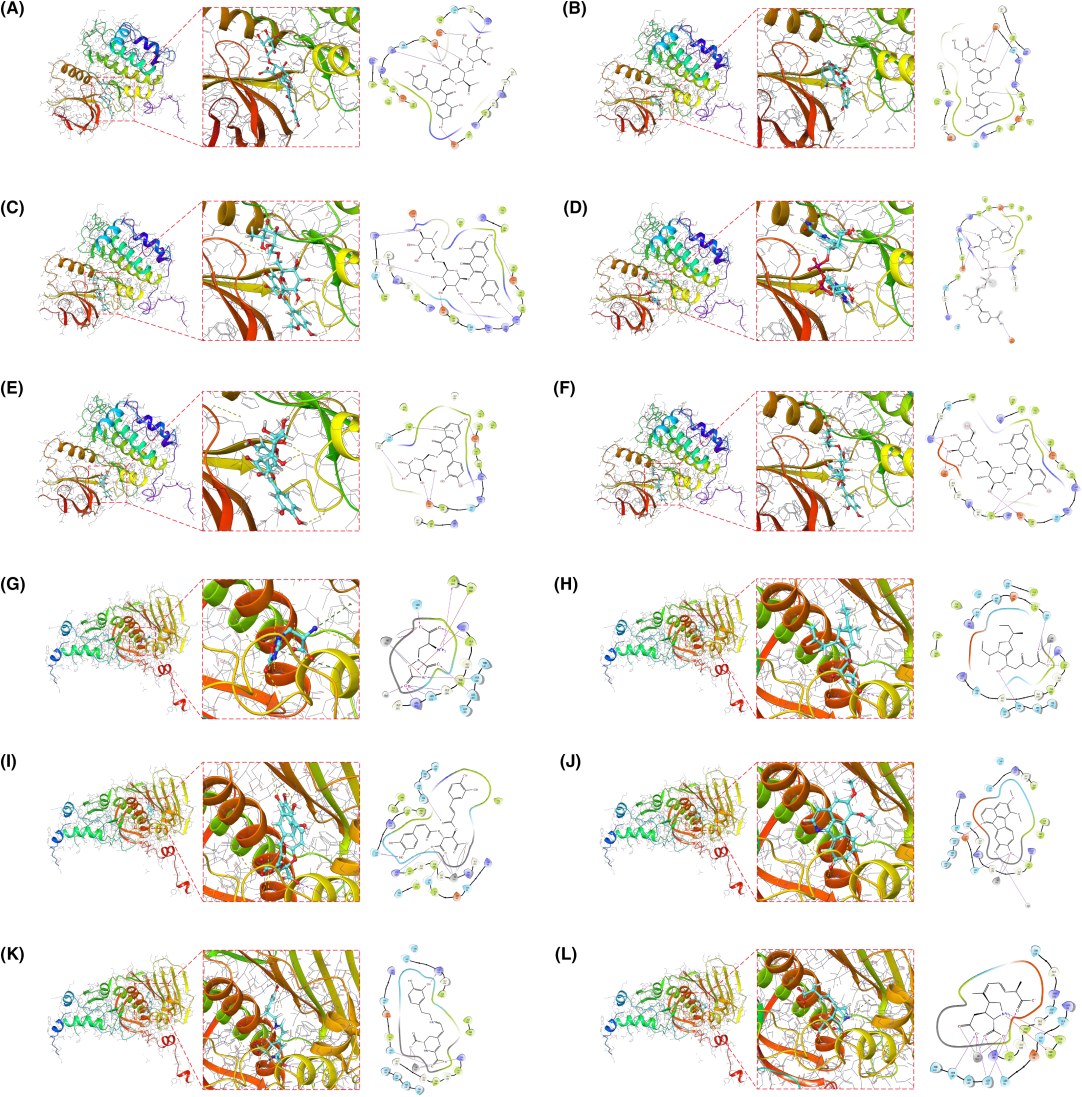
Molecular docking poses. The Figure shows the docking poses of the PLK1 active pocket with Nelumboside (A), Curculigoside C (B), Narcissoside (C), Fenugreekine (D), Isoquercetrin (E), and Epigeoside (F).The docking poses of the TOP2A active pocket with the QUISQUALIC ACID (G), Auxin b (H), Cichoric acid (I), Pareirubrine B (J), Miraxanthin V (K) and Domoic acid (L) in docking poses.

Molecular docking is a structure‐based computational algorithm for compound screening. We obtained the protein structures of PLK1 and TOP2A from the PDB database for molecular docking with natural small molecule compounds. The top six small molecules with the highest binding affinity to the PLK1 binding pocket (Nelumboside, Curculigoside C, Narcissoside, Fenugreekine, Isoquercetrin, and Epigeoside) and the top six small molecules with the highest binding affinity to the TOP2A binding pocket are shown, respectively (quisqualic acid, Auxin b, Cichoric acid, Pareirubrine B, Miraxanthin V and Domoic acid) (Figure 6). For example, Nelumboside forms hydrogen bonds with PLK1 amino acid residues Glu‐69, Arg‐136, Glu‐140, Asp‐194 and Lys‐178, where Glu‐69, Glu‐140 and Asp‐194 act as hydrogen bond acceptors, and Arg‐136 and Lys‐178 act as hydrogen bond donors. Narcissoside forms hydrogen bonds with PLK1 amino acid residues Glu‐131, Arg‐134, Arg‐136, Glu‐140, Gly‐180, Lys‐178 and Asp‐194, where Glu‐131, Arg‐134, Glu‐140, Gly‐180 and Asp‐194 act as hydrogen bond acceptors. Arg‐136 and Lys‐178 act as hydrogen bond donors.

### 3.9 Knockdown of MAPT inhibits HCC cell proliferation and migration

**FIGURE 7:**
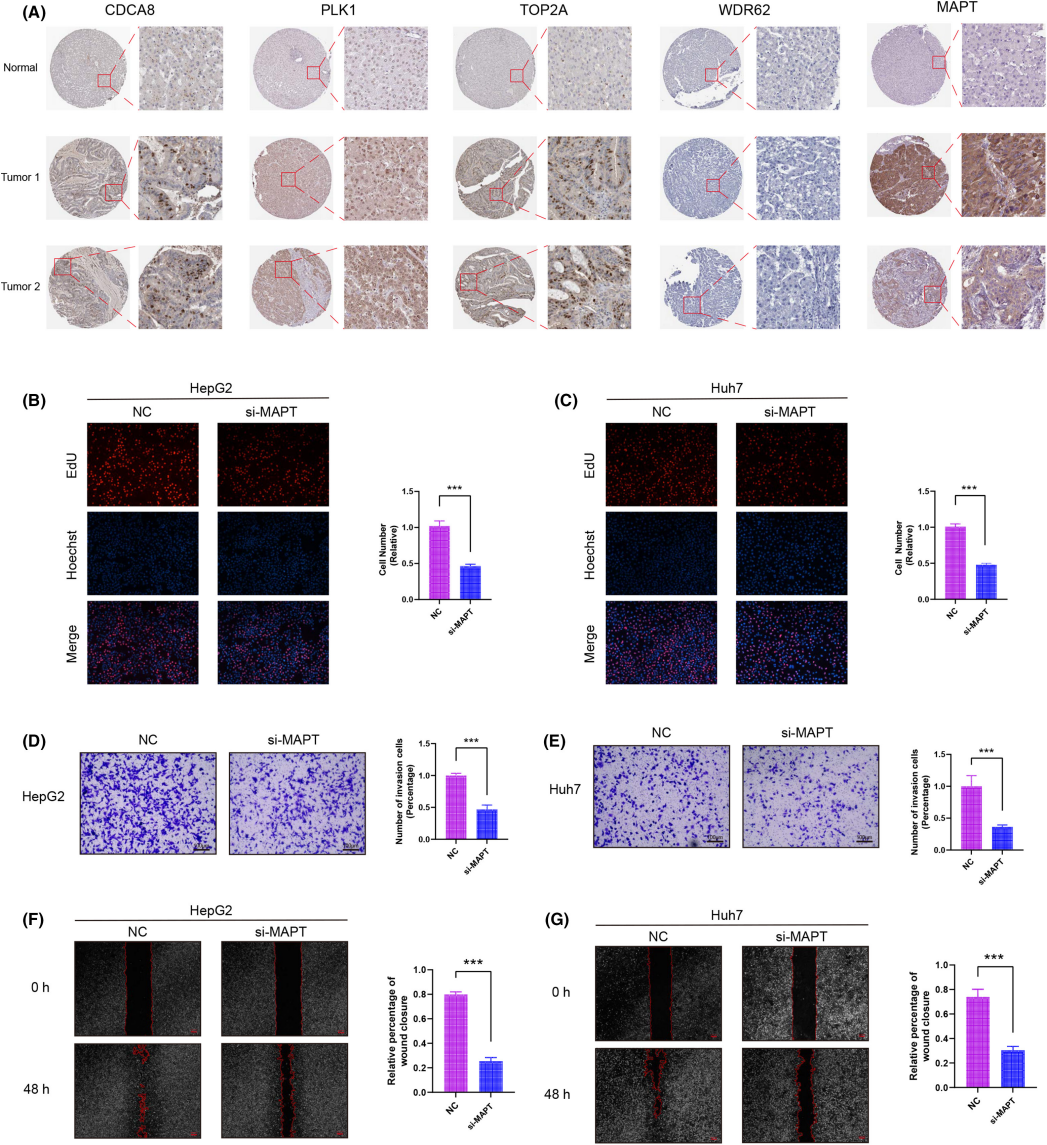
Knockdown of MAPT inhibited the proliferation and migration ability of hepatocellular carcinoma cells. (A) Immunohistochemical staining of MAPT, WDR62, PLK1, CDCA8 and TOP2A in liver normal tissues and hepatocellular carcinoma. EdU staining assay (B, C), transwell cell migration ability (D, E) and Wound‐healing assay (F, G) of HepG2 and Huh7 cells after transfection with si‐MAPT. Note *p < 0.05, **p < 0.01, ***p < 0.001.

We first compared the expression differences of MAPT, WDR62, PLK1, CDCA8 and TOP2A in normal hepatocyte tissues and HCC tissues by TCGA database, and the results showed that the expression levels of MAPT, WDR62, PLK1, CDCA8 and TOP2A were significantly higher in tumour cells than in normal tissues (Figure S1). Later we also verified the above results by immunohistochemical staining results of MAPT, WDR62, PLK1, CDCA8 and TOP2A in normal hepatocyte tissues and HCC tissues (Figure 7A).

We designed siRNA for MAPT to silence MAPT expression in human HCC cell lines HepG2 and Huh7 cells to investigate the role of MAPT in HCC. Plate cloning, Transwell and Wound‐healing experiments were performed by transfecting HepG2 and Huh7 cells with si‐MAPT, respectively. The results of EdU staining assay showed that the proliferation ability of HepG2 and Huh7 cells after knockdown of MAPT was significantly lower than that of the NC group (Figure 7B,C).The results of Transwell experiments showed that the cell migration ability of HEPG2 and HUH7 cells was significantly reduced after knockdown of MAPT (Figure 7D,E).The results of Wound‐healing experiments showed that after 48 h later, the migration ability of HepG2 and Huh7 cells in the si‐MAPT group was significantly lower than that in the NC group (Figure 7F,G).

## 4 DISCUSSION

More and more studies are now showing that liquid–liquid phase separation for targeting tumours is an important anticancer therapeutic strategy. Therefore, exploring the mechanism and function of liquid–liquid phase separation will provide some important insights for future cancer therapy.^18^, ^19^, ^20^ In this study, we constructed LLPSI based on five liquid–liquid phase separation genes (MAPT, WDR62, PLK1, CDCA8 and TOP2A). We found that LLPSI can be used as a marker for HCC staging and can effectively predict the prognosis and immunotherapy outcome of HCC patients, which was validated by external datasets. In addition, we identified natural small molecule drugs that can target liquid–liquid phase separation of core target proteins (PLK1 and TOP2A) by molecular docking, and finally verified that knockdown of MAPT can inhibit the proliferation and invasion of HCC by in vitro experiments.

The ability of a tumour to occur and develop is closely related to the fact that it can alter the tumour microenvironment in which it resides to help it evade immune surveillance.^21^ To clarify the relationship between LLPSI and the tumour microenvironment of HCC, we determined that patients in the high LLPSI group were closely associated with high levels of immune cell infiltration in the tumour microenvironment by using the ESTIMATE algorithm, GSEA enrichment analysis, ssGSEA algorithm and CIBERSORT algorithm. In addition, we compared the expression levels of immune checkpoints between the high/low LLPSI groups and found that patients in the high LLPSI group had significantly higher levels of immune checkpoints than those in the low LLPSI group, suggesting that the tumour cells in the high LLPSI group may have high immune escape potential. We predicted the immunotherapeutic effect of the high LLPSI group and low LLPSI group by TIDE scoring, and the results showed that patients in the high LLPSI group responded better to immunotherapy. On the other hand, we assessed the relationship between LLPSI and drug sensitivity by common chemotherapeutic agents. We found that LLPSI could distinguish the sensitivity of HCC to common chemotherapeutic agents. The above results suggest that LLPSI can assess the efficacy of immunotherapy and chemotherapeutic drugs in patients with HCC, which can provide a great help for the future treatment of HCC patients.

LLPSI is composed of five liquid–liquid phase separation genes including MAPT, WDR62, PLK1, CDCA8, and TOP2A. Microtubule‐Associated Protein Tau (MAPT) gene encodes the microtubule‐associated protein tau, which promotes microtubule assembly and stabilization and may be involved in the establishment and maintenance of neuronal polarity.^22^ In addition, the C‐terminus of the microtubule‐associated protein tau binds axonal microtubules, while the N‐terminus binds neural plasma membrane components, suggesting that tau acts as a connecting protein between the two.^22^, ^23^ WD Repeat Domain 62 (WDR62) is required for the development of the cerebral cortex, and plays a role in neuronal proliferation and migration.^24^ Cell Division Cycle Associated 8 (CDCA8) This gene encodes a component of the Chromosome Passenger Complex, which has an essential function at the mitophagy to ensure proper chromosome alignment and segregation and is required for chromatin‐induced microtubule stabilization and spindle assembly.^25^, ^26^ DNA Topoisomerase II Alpha (TOP2A) encodes a DNA topoisomerase, an enzyme that controls and alters the topological state of DNA during transcription. This ribozyme is involved in processes such as chromosome condensation, chromosome segregation, and relief of torsional stresses that occur during DNA transcription and replication.^27^, ^28^

There are some limitations to this study; first, the data for our analysis were obtained from public databases, which may have led to some case selection bias in case selection. In addition, although we collected several external datasets to validate the conclusions obtained in this study, it is still necessary to collect a large amount of clinical case data to further validate the accuracy of the results. Finally, further in vivo and in vitro experiments are needed to validate the specific mechanisms and functions of MAPT, WDR62, PLK1, CDCA8 and TOP2A in the liquid–liquid phase separation system of HCC.

## 5 CONCLUSION

In summary, we comprehensively analysed multiple aspects of HCC based on LLPSI constructed from liquid–liquid phase separation genes, and we found that LLPSI could effectively predict the prognosis and immunotherapy effect of HCC patients and validate them with external datasets. We also identified new prognostic and therapeutic biomarkers for HCC as well as targeted small molecule drugs from the perspective of liquid–liquid phase separation, which provides reliable clues for future precision treatment of HCC. In an era when immunotherapy holds great promise for cancer treatment, LLPSI provides guiding value for clinical diagnosis and individualized comprehensive treatment of HCC.

## AUTHOR CONTRIBUTIONS

**Zhiyong Wang:** Conceptualization (equal); data curation (equal); funding acquisition (equal); investigation (equal); methodology (equal); project administration (equal); validation (equal); visualization (equal); writing – original draft (equal). **Guoliang Wang:** Data curation (equal); formal analysis (equal); investigation (equal); methodology (equal). **Peng Zhao:** Data curation (equal); formal analysis (equal); funding acquisition (equal); investigation (equal); project administration (equal); supervision (equal); validation (equal); visualization (equal). **Ping Sun:** Formal analysis (equal); funding acquisition (equal); investigation (equal); software (equal); supervision (equal); visualization (equal); writing – review and editing (equal).

## FUNDING INFORMATION

This work was supported by the by the National Natural Science Foundation of China (No. 82002032 from Ping Sun, and No. 81700558 from Guoliang Wang).

## CONFLICT OF INTEREST STATEMENT

The authors declare no conflicts of interest.

## Supporting information


Figure S1.

## Data Availability

All data utilized in this study are included in this article and all data supporting the findings of this study are available on reasonable request from the corresponding author.
