# Supplementary figures and images for: The liquid–liquid phase separation signature predicts the prognosis and immunotherapy response in hepatocellular carcinoma
Source: J Cell Mol Med. 2024 Jul 28;28(14):e18446. doi: 10.1111/jcmm.18446 (PMC11284120; doi:10.1111/jcmm.18446)

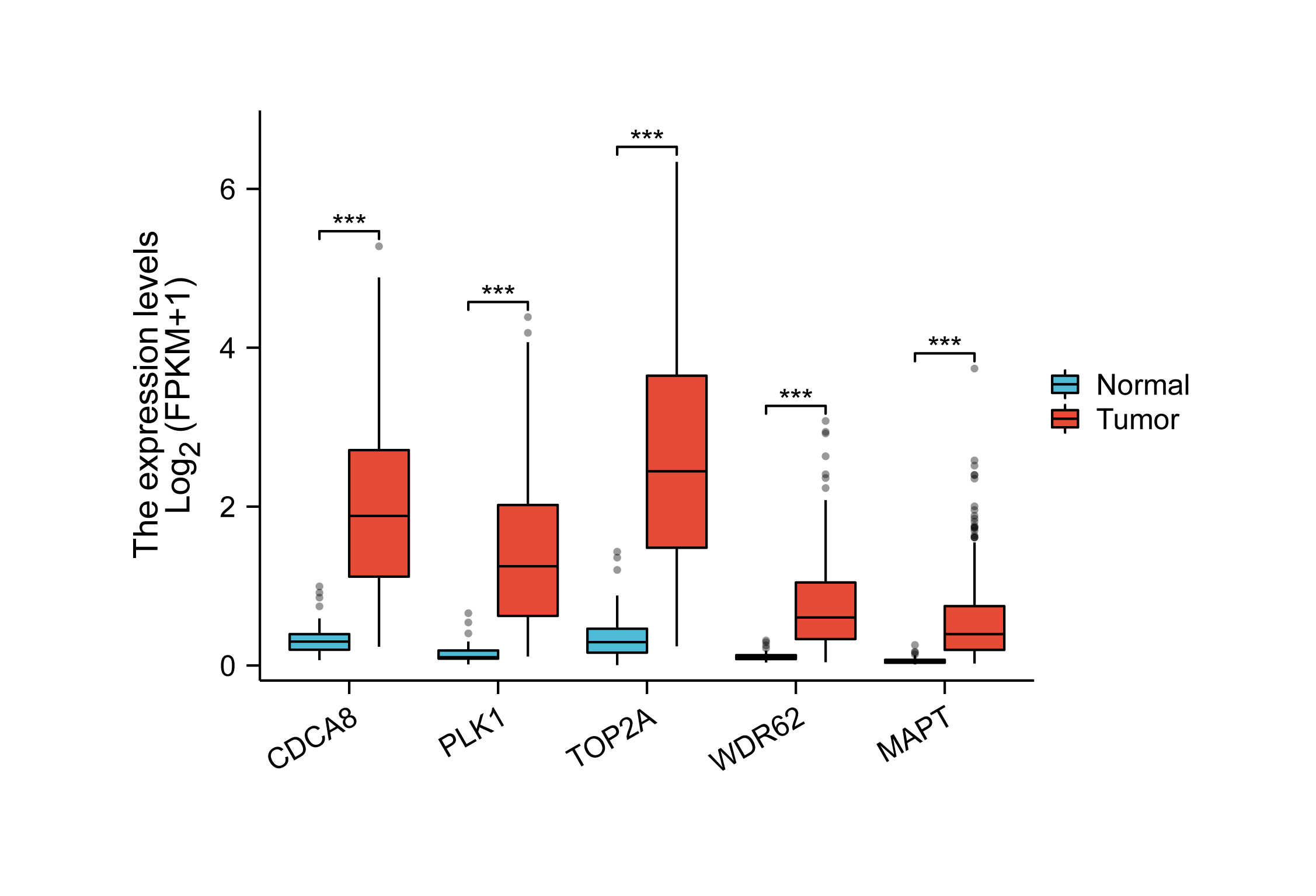

Supplement: Supplementary file 1 — Figure S1. [file JCMM-28-e18446-s001.tif]
